# A Two-Headed Monster to Avert Disaster: HBS1/SKI7 Is Alternatively Spliced to Build Eukaryotic RNA Surveillance Complexes

**DOI:** 10.3389/fpls.2018.01333

**Published:** 2018-09-12

**Authors:** Jacob O. Brunkard, Barbara Baker

**Affiliations:** ^1^Department of Plant and Microbial Biology, University of California, Berkeley, Berkeley, CA, United States; ^2^Plant Gene Expression Center, USDA Agricultural Research Service, Albany, CA, United States

**Keywords:** RNA exosome, innate immunity, antiviral defense, RNA interference, alternative splicing, HBS1, SKI7

## Abstract

The cytosolic RNA exosome, a 3′→5′ exoribonuclease complex, contributes to mRNA degradation in eukaryotes, limiting the accumulation of poorly-translated, improperly translated, or aberrant mRNA species. Disruption of cytosolic RNA exosome activity allows aberrant RNA species to accumulate, which can then be detected by host antiviral immune systems as a signature of pathogen infection, activating antiviral defenses. SKI7 is a critical component of the cytosolic RNA exosome in yeast, bridging the catalytic exoribonuclease core with the SKI2/SKI3/SKI8 adaptor complex that guides aberrant RNA substrates into the exosome. The ortholog of *SKI7* was only recently identified in humans as an alternative splice form of the *HBS1* gene, which encodes a decoding factor translational GTPase that rescues stalled ribosomes. Here, we identify the plant orthologs of *HBS1/SKI7*. We found that HBS1 and SKI7 are typically encoded by alternative splice forms of a single locus, although some plant lineages have evolved subfunctionalized genes that apparently encode only HBS1 or only SKI7. In all plant lineages examined, the *SKI7* gene is subject to regulation by alternative splicing that can yield unproductive transcripts, either by removing deeply conserved SKI7 coding sequences, or by introducing premature stop codons that render *SKI7* susceptible to nonsense-mediated decay. Taking a comparative, evolutionary approach, we define crucial features of the SKI7 protein shared by all eukaryotes, and use these deeply conserved features to identify SKI7 proteins in invertebrate lineages. We conclude that SKI7 is a conserved cytosolic RNA exosome subunit across eukaryotic lineages, and that *SKI7* is consistently regulated by alternative splicing, suggesting broad coordination of nuclear and cytosolic RNA metabolism.

## Introduction

Viruses dominate the biosphere, massively outnumbering cellular organisms (Koonin, 2017). Unlike cellular organisms, however, all viruses are obligate parasites that depend on organismal ribosomes for translation and replication (Walsh et al., 2013; Wang, 2015). Viral hosts are under strong selective pressure to recognize and limit viral parasitism, and in parallel, viruses are under strong selective pressure to evade host surveillance mechanisms and coopt the host translation machinery (Walsh et al., 2013; Molleston and Cherry, 2017). Host organisms have evolved various defense and immune systems to protect against viruses, including several approaches to detect non-host (viral) nucleic acids and then trigger defenses and guided destruction of viral genomes (Barrangou et al., 2007; Narayanan and Makino, 2013; Szittya and Burgyán, 2013; Abernathy and Glaunsinger, 2015; Li et al., 2015; Molleston and Cherry, 2017). A famous example is the evolution of bacterial “CRISPR-Cas” systems that incorporate viral dsDNA into short palindromic repeats in the host genome, and then transcribe these incorporated DNA sequences into guides that target DNases to specifically cleave complementary viral DNA (Brouns et al., 2008). Eukaryotes have evolved two major approaches to detect viral nucleic acids and mount immune responses. One of these, the interferon system, evolved in the *Gnathostomata* lineage (vertebrates with jaws) (Zou et al., 2009). In these organisms, pattern recognition receptors detect aberrant nucleic acids (such as dsRNA) and then rapidly activate transcription of a class of cytokines, called interferons, that are secreted, detected by cell surface receptors (of both infected and uninfected cells), and ultimately trigger transcriptional reprogramming that limits viral replication and spread (Li et al., 2015; Rigby and Rehwinkel, 2015). Outside the *Gnathostomata* lineage, most eukaryotes employ RNA interference (RNAi) systems to combat viral infections (Shabalina and Koonin, 2008).

RNA interference relies on endoribonucleases of the RNase III family that recognize and cleave double-stranded RNA (dsRNA) as basal defense against viral infection (Seo et al., 2013; Nicholson, 2014). RNase III may recognize dsRNA synthesized by a viral replicase, or the host may synthesize dsRNA from viral RNA templates using an endogenous RNA-dependent RNA polymerase; in either case, the dsRNA is processed by RNase III (Blevins et al., 2006; Szittya and Burgyán, 2013). In plants, two RNase III enzymes, called DICER-LIKE 4 (DCL4) and DCL2, are primarily responsible for defense against RNA viruses (Szittya and Burgyán, 2013; Andika et al., 2015). Cleavage of viral dsRNA by DCL4/DCL2 generates viral short interfering RNAs (vsiRNAs). vsiRNAs are loaded into ARGONAUTE (AGO) proteins in RNAi complexes (RISCs) that search for further, complementary viral RNAs using the 21- or 22-nt siRNA sequence. Any RNA recognized by the RISC, presumably a viral genome, is then cleaved and degraded. In a poorly understood process, RDR6 can be recruited to the cleaved viral RNA to synthesize another dsRNA template for processing by DCL4/DCL2 (Qu et al., 2005, 2008; Qi et al., 2009). This RDR6-dependent process can amplify the number of antiviral siRNAs available for the immune system (Garcia-Ruiz et al., 2010).

The DCL4/DCL2/RDR6 surveillance system has since been coopted in the plant lineage to regulate endogenous gene expression (Peragine et al., 2004; Allen et al., 2005; Cuperus et al., 2010). After cleavage by a 22-nt miRNA (or, in exceptional cases, some 21-nt miRNAs), endogenous transcripts can become templates for copying by RDR6 and cleavage by DCL4/DCL2 into siRNAs (Allen et al., 2005). These siRNAs can then amplify silencing of the miRNA target by guiding RISCs to multiple sites in transcripts with the original miRNA target, or may act *in trans* by guiding the RISC to other transcripts with complementary (or nearly complementary) ∼21-nt sequences. The DCL4/DCL2/RDR6 system is used by plants to regulate diverse biological processes, including auxin-mediated developmental patterning (Peragine et al., 2004; Vazquez et al., 2004; Fahlgren et al., 2006) and suppression of disease resistance *R* genes (Li et al., 2012; Deng et al., 2018), whose overexpression can cause autoimmune syndromes (Yi and Richards, 2009).

After cleavage by RISC endonucleases, viral RNA must be degraded by host RNA exoribonucleases (Abernathy and Glaunsinger, 2015). Two major exoribonuclease mechanisms are conserved across eukaryotes: 5′→3′ RNA degradation by the EXORIBONUCLEASEs (XRNs) (Nagarajan et al., 2013) and 3′→5′ RNA degradation by the RNA exosome (Zinder and Lima, 2017). These enzymes are involved in housekeeping degradation of host mRNAs in the cytosol and in processing RNA transcripts in the nucleus. The XRNs have evolved two distinct gene families to handle these processes: the *XRN1* family encodes a cytosolic enzyme, and the *XRN2* family encodes a nuclear enzyme. In an ancestor of plants, an *XRN2* paralog evolved cytosolic localization and the *XRN1* gene lineage was lost; the plant cytosolic XRN is therefore called *XRN4*, but is functionally equivalent to eukaryotic *XRN1* (Nagarajan et al., 2013). The RNA exosome forms a large, multiprotein complex, and localizes to both the cytosol and nucleus. Adaptor complexes then guide RNA substrates to the RNA exosome; for example, the nuclear TRAMP complex guides ribosomal RNA (rRNA) and small nucleolar RNA (snoRNA) to the RNA exosome for processing (Tollervey, 2015). In the cytosol, the RNA exosome is chaperoned to substrates by the SKI2/SKI3/SKI8 complex (Schmidt et al., 2016), which facilitates degradation of cleaved RNA (including deadenylated mRNAs) by guiding these RNAs into the RNA exosome catalytic core.

During RNAi, the SKI2/SKI3/SKI8 complex is recruited to siRNA-cleaved mRNAs by stalled ribosomes that reach the cleavage site (Orban and Izaurralde, 2005; Branscheid et al., 2015; Zhang et al., 2015; Szadeczky-Kardoss et al., 2018). Stalled ribosomes at the cleavage site are recognized by PELOTA/DOM34 (a translational decoding factor) and HBS1 (a translational GTPase), which recruit SKI2/SKI3/SKI8 and the RNA exosome to degrade the 5′ fragment of the cleaved mRNA (Orban and Izaurralde, 2005). Supporting this hypothesis, loss of PELOTA, HBS1, or SKI2 stabilizes the 5′ fragment of transcripts cleaved in their open reading frames by miRNA-guided RISCs, in both *Drosophila* and plants (Orban and Izaurralde, 2005; Szittya and Burgyán, 2013; Branscheid et al., 2015; Hashimoto et al., 2017; Szadeczky-Kardoss et al., 2018). Extending this model, we propose that PELOTA, HBS1, SKI2/SKI3/SKI8, and SKI7 are likely necessary for degradation of messenger viral RNAs after cleavage by vsiRNAs. Moreover, PELOTA, HBS1, SKI2/SKI3/SKI8, and SKI7 are all proposed to contribute to degradation of transcripts with premature termination codons via nonsense-mediated decay (NMD) (Mitchell and Tollervey, 2003; Takahashi et al., 2003; Arribere and Fire, 2018). Structural features of viral RNA can be recognized as nonsense transcripts by eukaryotic cells, leading to viral RNA degradation by NMD. Thus, PELOTA, HBS1, and SKI7 could have antiviral roles by participating in NMD. In tomato, a natural loss-of-function variant of *pelota*, called *ty-5*, confers resistance to *Tomato yellow curly leaf virus* (TYCLV), highlighting the importance of the PELOTA/HBS1 RNA degradation machinery in plant-virus interactions (Lapidot et al., 2015).

In humans and in plants, the SKI2 has been implicated in preventing endogenous RNA from triggering immune responses. Depletion of the human SKI2 ortholog, HsSKIV2L (Kalisiak et al., 2017), allows accumulation of aberrant RNA species that are sensed by nucleic acid pattern recognition receptors, which in turn activate type I interferon expression and trigger autoimmune/autoinflammatory responses (Eckard et al., 2014; Rigby and Rehwinkel, 2015). Loss-of-function mutations in *SKIV2L* have been genetically linked to autoimmune syndromes (Crow et al., 2006), including systemic lupus erythematosus (Crow et al., 2006), and this association with autoimmune syndromes may be related to its role in limiting autoinflammatory responses to endogenous RNAs (Eckard et al., 2014). In *Arabidopsis thaliana*, disruption of the *SKI2* gene has a similar effect: deadenylated RNA species accumulate in the cytosol (e.g., transcripts cleaved by miRNAs), and become available as templates for RDR6 to generate dsRNA (Branscheid et al., 2015), a process that is comparable to RDR6 copying of cleaved viral RNA. These RDR6-dependent dsRNA molecules are subsequently processed by DCL4/DCL2, generating siRNAs that silence host gene expression. Thus, in both humans and plants, SKI2 is required to limit the accumulation of aberrant RNA species that are otherwise detected by the cell as potential viruses, triggering antiviral immune responses in the absence of pathogen attack.

Recently, structural studies of the cytoplasmic RNA exosome in yeast and humans have revealed the crucial importance of SKI7 in bridging the RNase exosome complex with the SKI2/3/8 adaptor complex that feeds cytosolic RNA substrates into the exosome (Kalisiak et al., 2017). *SKI7* was first identified in the same genetic screen as the other cytoplasmic RNA exosome components, but unlike *SKI2*, *SKI3*, and *SKI8*, orthologs of *SKI7* were not readily identifiable in other eukaryotic genomes. A genomic investigation of *Lachancea kluyveri*, a fungus closely related to *S. cerevisiae*, revealed that SKI7 is encoded by an alternative splice form of the *HBS1* locus in that species (Marshall et al., 2013). In *S. cerevisiae*, *HBS1* and *SKI7* are functionally distinct homeologs that derive from a whole-genome duplication in a recent ancestor of *S. cerevisiae*; *L. kluyveri* diverged from this lineage shortly before the whole-genome duplication. The authors of this study noted briefly that the *HBS1* locus is potentially alternatively spliced in other eukaryotic lineages, but did not systematically identify *HBS1/SKI7* orthologs in metazoans or plants. Subsequent studies revealed that the vertebrate *HBS1* locus is also alternatively spliced, and that one of these splice forms, *HBS1Lv3*, encodes a protein that serves the same function as SKI7 in *S. cerevisiae* (Kalisiak et al., 2017).

**FIGURE 1:**
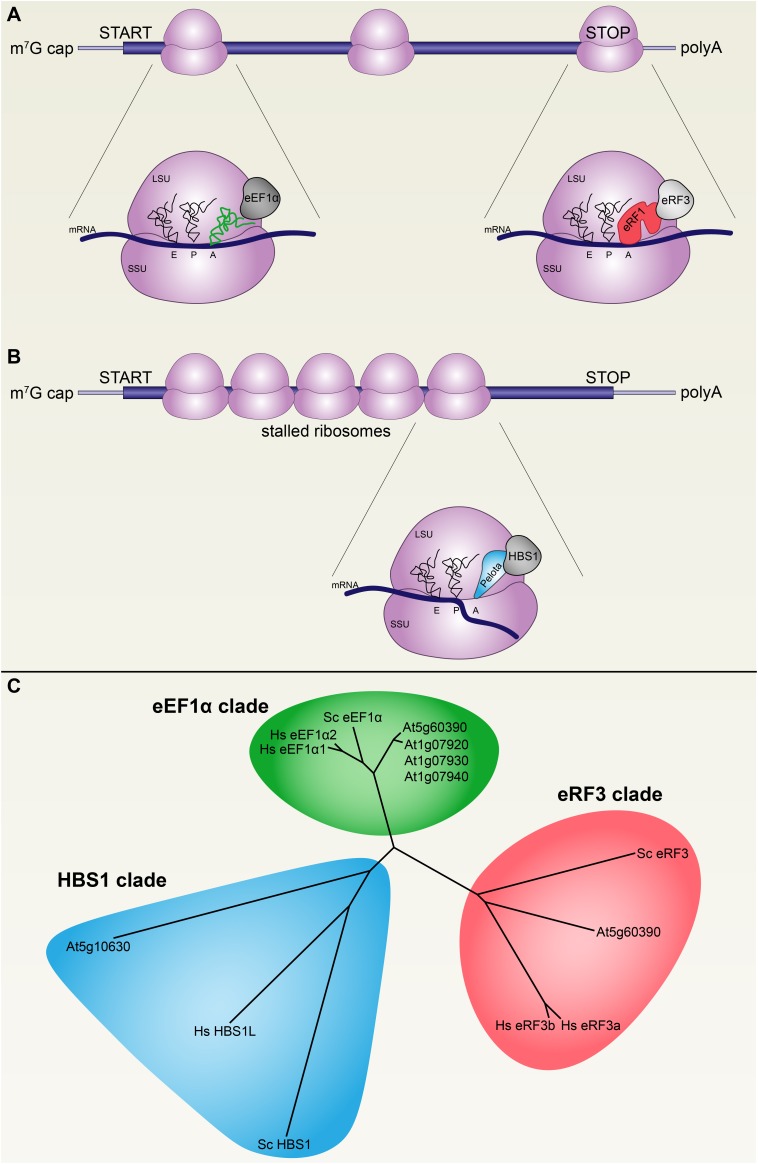
Decoding translational GTPases are conserved across eukaryotes. **(A)** During active translation, mRNA codons are decoded at the aminoacyl site (A site) of 80S ribosomes (purple) by cognate tRNAs (green) in complex with the GTPase eukaryotic translation elongation factor 1 alpha (eEF1α, gray). Stop codons are recognized at the A site by eukaryotic release factor 1 (eRF1) in complex with the GTPase eukaryotic release factor 3 (eRF3), which terminates translation and releases ribosomes. **(B)** Stalled ribosomes are recognized by PELOTA in complex with the GTPase HBS1, which triggers premature ribosome release and degradation of the mRNA and the nascent polypeptide. Ribosome may stall on mRNAs with complex secondary structures or after endonucleolytic cleavage by RNAi silencing complexes, among other possible causes. **(C)** The three decoding factor translational GTPases are conserved across the plant, fungal, and metazoan lineages, as represented in this phylogeny by protein sequences from *Arabidopsis thaliana*, *Saccharomyces cerevisiae*, and *Homo sapiens*, respectively.

**FIGURE 2:**
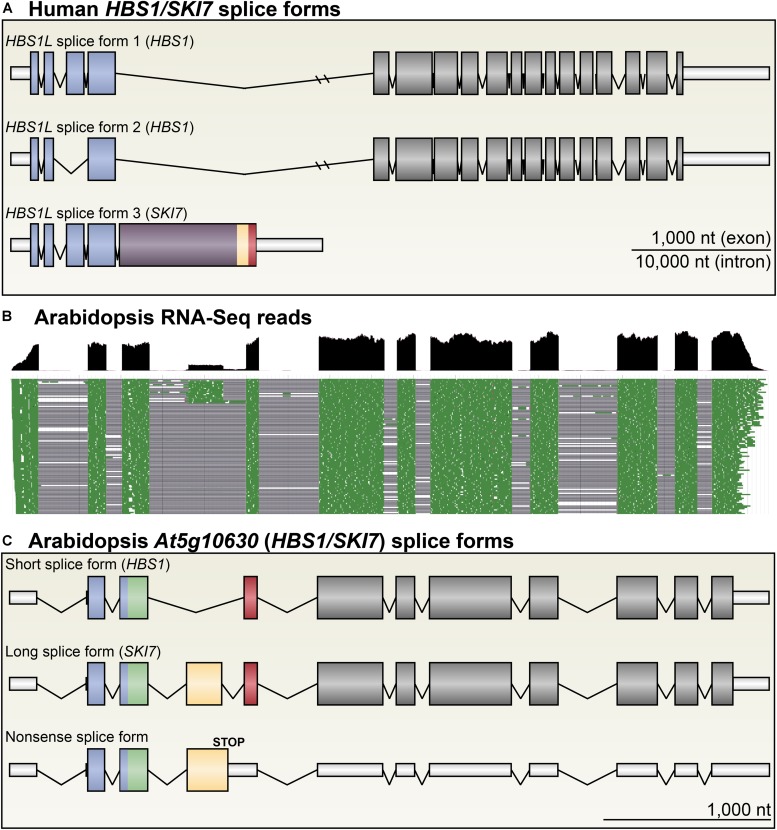
Arabidopsis *HBS1/SKI7* is alternatively spliced. **(A)** The human *HBS1/SKI7* locus, called *HBS1L*, produces three primary splice forms: *HBS1L* splice form 1, a long splice form with 18 exons; *HBS1L* splice form 2, a slightly shorter splice form skipping exon 3; and *HBS1L* splice form 3, which selects an alternative exon 5 followed by transcription termination. *HBS1Lv3* includes sequences that promote interaction of the protein with the RNA exosome (yellow and red; see **Figure 4A**). **(B)** RNA-Seq of light-grown seedlings shows that *At5g10630* is alternatively spliced into three major splice forms (Cheng et al., 2017). Cumulative RNA-Seq reads are shown in black (top panel), and select individual aligned reads are shown in green (bottom panel), with spliced sequences indicated by a black line. Note that reads for the fourth exon are ∼20% of the level of reads for the other coding sequence exons. **(C)** *At5g10630* forms three major splice forms. A short splice form (top) skips exon 4 (yellow), yielding a transcript that encodes HBS1 **(A)**. A long splice form (middle) includes exon 4 (yellow), yielding a transcript that encodes SKI7 **(A)**. Rarely, an alternative acceptor site is selected for exon 4, adding five amino acids with no apparently functional consequence. A nonsense splice form (bottom) retains intron 4, which includes two codons, yielding a transcript that is likely subject to NMD. Exons are colored to match protein models in subsequent figures; UTRs are indicated with narrow, white bars.

The discovery that SKI7 and HBS1 are encoded by the same locus in many fungi and vertebrates is perhaps surprising because of their apparently unrelated functions. HBS1 is a translational GTPase that is required for the release of stalled ribosomes from mRNA, along with its interacting partner, PELOTA/DOM34 (**Figure 1**; Shao et al., 2016). SKI7 is instead a bridge between the RNA exosome and the SKI2 complex, and while the *S. cerevisiae* SKI7 has a C-terminal HBS1-like GTPase domain, this terminus is dispensable for its functions (Horikawa et al., 2016). Highlighting their distinct functions, the two protein isoforms encoded by the *HBS1/SKI7* locus in *Lachancea kluyveri* can complement only one of the *S. cerevisiae* Δ*hbs1* or Δ*ski7* mutant strains: the long, SKI7-like isoform only complements Δ*ski7*, and the shorter, HBS1-like isoform only complements Δ*hbs1* (Marshall et al., 2013). In humans, the isoform encoding the functional ortholog of SKI7, HBS1Lv3, loses exons that encode the entire C-terminal HBS1 GTPase domain (which is essential for HBS1 functions), and instead gains an exon that encodes an RNA exosome-interacting surface (**Figure 2A**). It remains unclear why vertebrate and most fungal genomes would retain a single locus to encode both HBS1 and SKI7, whereas *S. cerevisiae* has successfully evolved two distinct loci to separate these functions. Here, we take advantage of these recent insights into the gene and protein structures of *HBS1/SKI7* in other eukaryotes to identify and characterize *HBS1/SKI7* orthologs in the plant lineage.

## Results

### Identification of *HBS1/SKI7* in *A. thaliana*

Transcripts are decoded during translation by duplexes composed of a translational GTPase (trGTPase) and either an aminoacyl-tRNA or a ribosome release factor (Dever and Green, 2012; Shao et al., 2016). There are three major classes of these decoding trGTPases: eEF1α (eukaryotic Elongation Factor 1 alpha), which mediates delivery of aminoacyl-tRNAs to the 80S ribosome; eRF3 (eukaryotic Release Factor 3), which mediates delivery of eRF1 (eukaryotic Release Factor 1) to stop codons to terminate translation and facilitate ribosome dissociation from transcripts; and HBS1 (Hsp80 subfamily B Suppressor 1), which mediates delivery of PELOTA (a.k.a. Dom34 in yeast, Duplication Of Multilocus region) to stalled ribosomes to terminate translation and facilitate ribosome dissociation (**Figures 1A,B**) (Carr-Schmid et al., 2002; Becker et al., 2012; Shao et al., 2016; Hashimoto et al., 2017). Since all decoding trGTPases are similar to each other, we began by identifying orthologs of each of the three decoding factor trGTPases (eEF1α, eRF3, and HBS1) in *Arabidopsis thaliana* in order to confidently distinguish plant HBS1 from the other trGTPases (**Figure 1C** and **Supplementary Data File 1**). Separate loci encode orthologs of these proteins that localize to mitochondria and/or plastids; these were removed from our analysis to focus only on cytoplasmic proteins. Arabidopsis encodes one copy of eRF3 (At1g18070), four copies of eEF1α (At5g60390 and three tandem paralogs, At1g07920, At1g07930, and At1g07940), and one copy of HBS1 (At5g10630). The three tandem *eEF1*α paralogs evolved recently (this complex locus is not conserved across *Brassicaceae*). All of the decoding trGTPases are expressed throughout Arabidopsis development, although it should be noted that *eRF3* transcripts are about one order of magnitude more abundant than *HBS1* transcripts, and *eEF1*α transcripts are at least two orders of magnitude more abundant than *HBS1* transcripts, consistent with their distinct roles in translation (Cheng et al., 2017).

**FIGURE 3:**
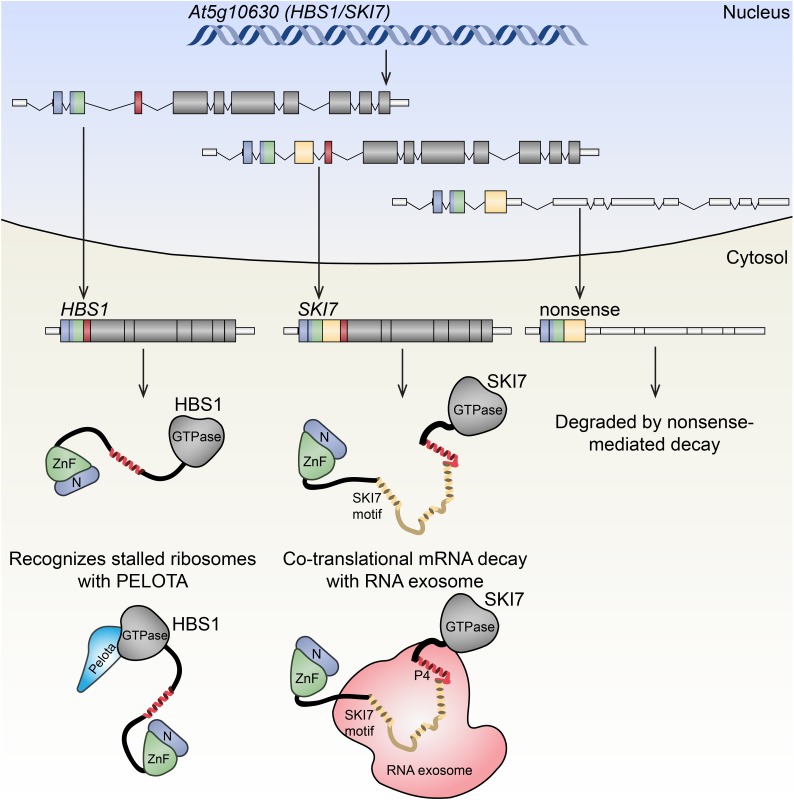
Alternative splicing of Arabidopsis *HBS1/SKI7* has functional consequences. Arabidopsis *HBS1/SKI7* (*At5g10630*) is alternatively spliced in the nucleus, generating at least three distinct transcripts that are exported to the nucleus. The *HBS1* transcript (left) putatitively encodes a translational GTPase, HBS1, that interacts with the decoding factor PELOTA to recognize stalled ribosomes. The *SKI7* transcript (center) putatitively encodes SKI7, a protein that associates with the cytosolic RNA exosome, a 3′→5′ exoribonuclease complex that degrades aberrant transcripts. A third splice form (right) encodes a premature translation codon in the fourth exon, presumably triggering NMD after a pioneer round of translation.

**FIGURE 4:**
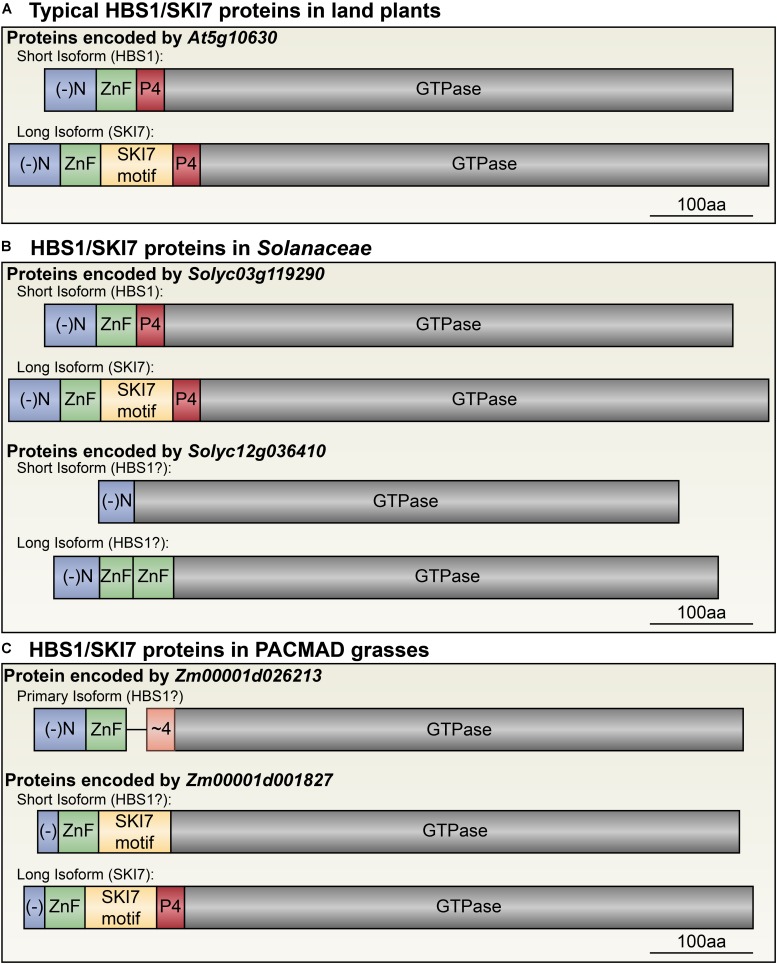
Diversity of HBS1/SKI7 isoforms among plant lineages. **(A)** The typical plant *HBS1/SKI7* locus (here exemplified by *At5g10630*, but also found in *P. patens*, *A. trichocarpa*, *T. cacao*, *M. truncata*, *N. benthamiana*, and *S. lycopersicum*, among other species) encodes two protein isoforms, HBS1 (top) and SKI7 (bottom), defined by the inclusion or exclusion of the SKI7-like motif (yellow). **(B)** The *Solanaceae* have two distinct *HBS1/SKI7*-like loci: one is similar to the typical plant *HBS1/SKI7* locus in panel a (exemplified by *Solyc03g119290*), and the second encodes only an HBS1-like protein, and is alternatively spliced to include or exclude two Zn finger domains (exemplified by *Solyc12g036410*). **(C)** In the PACMAD grasses, one *HBS1/SKI7*-like locus encodes an HBS1-like protein lacking the SKI7-like motif and with a poorly-aligned Patch 4-like motif (exemplified by *Zm0001d026213*). A second *HBS1/SKI7*-like locus (exemplified by *Zm0001d001827*) can encode two proteins: a long SKI7 isoform and a short isoform that excludes the Patch 4-like motif, and thus may function as an HBS1-like protein or be a loss-of-function isoform of SKI7. (-)N, blue, negatively charged N-terminus; ZnF, green, RAN2-type Zinc finger domain; SKI7 motif, yellow, the SKI7-like motif often encoded by a cassette exon; P4, red, the Patch 4-like motif; ∼4, orange, a poorly-aligned Patch 4-like motif unique to grass HBS1 isoforms; GTPase, gray, the HBS1/SKI7 decoding factor translational GTPase domain.

The Arabidopsis *At5g10630* (*HBS1*) locus is annotated with several different possible transcripts, but there are only two major protein isoforms predicted to be encoded by these transcripts: a long splice form (**Figure 2C**) encodes a protein that is 738 amino acids long (**Figures 3**, **4A**), and a short splice form (**Figure 2C**) skips an exon to encode a shorter protein that is 668 amino acids long (**Figures 3**, **4A**). High-throughput sequencing of RNA from light-grown seedlings shows that the alternative cassette exon is included in approximately 20% of *At5g10630* transcripts (**Figure 2B**) (Cheng et al., 2017). A very minor splice form uses a weak 3′ splice site that adds 15nt to the 5′ end of the cassette exon; this is included in at most 4% of *At5g10630* transcripts in light-grown seedlings. Because this minor splice form is rare and does not cause significant changes in the protein sequence (it neither induces a frame-shift nor includes a stop codon, and only adds 5 amino acids to the protein), it was not investigated any further. Another very minor splice form (also at most 4% of *At5g10630* transcripts in light-grown seedlings, **Figure 2B**) retains the intron at the 3′ end of the cassette exon (**Figures 2C**, **3**), which introduces two in-frame stop codons (this retained intron can thus be considered a “poison intron”). Since these stop codons are upstream of several exon-exon junctions, this splice form is presumably subject to NMD, and is any case unproductive. We confirmed the RNA sequences of these four splice forms of *At5g10630* by RT-PCR using primers surrounding the alternatively spliced exons, followed by TOPO cloning and Sanger sequencing.

### Structural Features of the Arabidopsis HBS1/SKI7 Protein Isoforms

**FIGURE 5:**
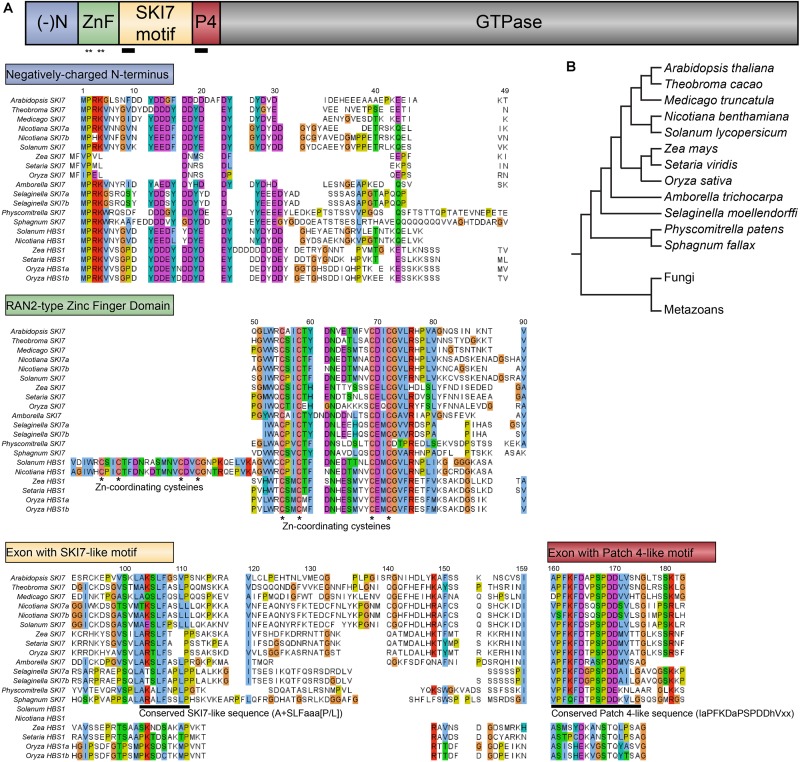
Evolution of SKI7-like amino acid sequences illustrated in select plant species. **(A)** Select SKI7-like proteins encoded by *SKI7/HBS1* loci and HBS1-like proteins encoded by exclusively *HBS1* loci are aligned to illustrate conserved and divergent sequences. Amino acids are colored following the Clustal standard (cyan – hydrophobic; red – positively charged; magenta – negatively charged; green – polar; cyan – aromatic; coral – cysteines; orange – glycines; yellow – prolines), with poorly conserved amino acids left uncolored. The negatively charged N-terminus (top) is highly similar across all proteins, although the grass SKI7 orthologs encode a much shorter N-terminus. The RAN2-type Zinc finger domain (middle) is highly similar in all plant species, including universal conservation of the Zn-coordinating cysteines (indicated with asterisks). This domain has been duplicated in the *Solanaceae HBS1* loci. The cassette exons that encode the SKI7-like motif and the Patch 4-like motif (bottom) are largely well-conserved in SKI7 proteins (deeply conserved eukaryotic sequences indicated with a black line; see **Figure 4**). *Solanaceae* HBS1-like proteins have lost both of these motifs. Grass HBS1-like proteins have lost the SKI7-like motif and much of the Patch 4-like motif. **(B)** Evolutionary relationships among the species included in this figure are depicted by this cladogram.

Comparative analysis of the two proteins predicted to be encoded by Arabidopsis *HBS1/SKI7* with the functionally characterized orthologs of *HBS1* (human *HBS1Lv1*, baker’s yeast *Hbs1*, and budding yeast *Lachancea kluyveri HBS1*) and *SKI7* (human *HBS1Lv3*, baker’s yeast *Ski7*, and budding yeast *L. kluyveri SKI7*) allowed us to identify several conserved regions in the Arabidopsis proteins (**Figures 4A**, **5A**). The N-terminus (aa 1–50) begins with a stretch of mostly negatively charged amino acids (40% of the first 50 amino acids are aspartic acid or glutamic acid, **Figure 3**). This is followed by a single Zinc finger domain of the RanBP2 superfamily (aa 51–75, **Figure 5A**), putatively involved in protein-protein interactions. If the transcript is alternatively spliced to include a cassette exon, the next region encodes an amino acid sequence with some similarity to the polypeptide encoded by the *L. kluyveri* alternative exon that determines SKI7 functionality (Marshall et al., 2013). This region includes a motif that corresponds to the HsSKI7 RxxxFxxxL motif required for recruiting HsSKI7 to the RNA exosome (Kalisiak et al., 2017). We have named this the “SKI7-like motif” (**Figure 5A**). Immediately after the sequence encoded by the cassette exon is the “Patch 4-like” motif (**Figure 5A**), named after the homologous yeast sequence, which was dubbed the “patch 4” motif (Kowalinski et al., 2016). In the human SKI7 protein, this is called the “PFDFxxxSPDDIVKxNQ motif” (Kalisiak et al., 2017). The Patch 4-like motif is found in all SKI7 proteins, but is not conserved in HBS1 proteins, and is proposed to mediate interactions between SKI7 and the RNA exosome subunit Csl4. Finally, the remaining C-terminus of the At5g10630 protein is an HBS1-like translational GTPase (**Figure 4A**). Thus, we defined the archetypical plant HBS1/SKI7 protein with five regions: a negatively charged N-terminus, a Zn-finger domain, a SKI7-like motif, a Patch 4-like motif, and the C-terminal GTPase (**Figure 4A**).

### Evolution of the *HBS1/SKI7* Locus in Land Plants

We next used the Arabidopsis *HBS1/SKI7* locus to search for orthologs in the genomes of land plants. We included the following species (**Figure 5B**): *Sphagnum fallax* and *Physcomitrella patens* (two distantly related species representing the moss lineage, which diverged early during land plant evolution), *Selaginella moellendorffi* (representing the lycophyte lineage, which diverged early during tracheophyte evolution), *Amborella trichopoda* (an ancient lineage of earliest-diverging flowering plants), *Solanum lycopersicum* and *Nicotiana benthamiana* (asterid eudicots in the *Solanaceae* family), *Medicago truncatula* (a rosid eudicot in the fabid order), *Theobroma cacao* (a close relative of *Arabidopsis* in the malvid order of rosid eudicots), *Oryza sativa* (a species of the BOP clade of grasses), and *Zea mays* and *Setaria viridis* (panicoid grasses). We focused on this limited set of taxa because of their excellent genome and transcriptome sequences, which allowed us to confidently identify potential alternative splice forms of the *HBS1/SKI7* locus.

**FIGURE 6:**
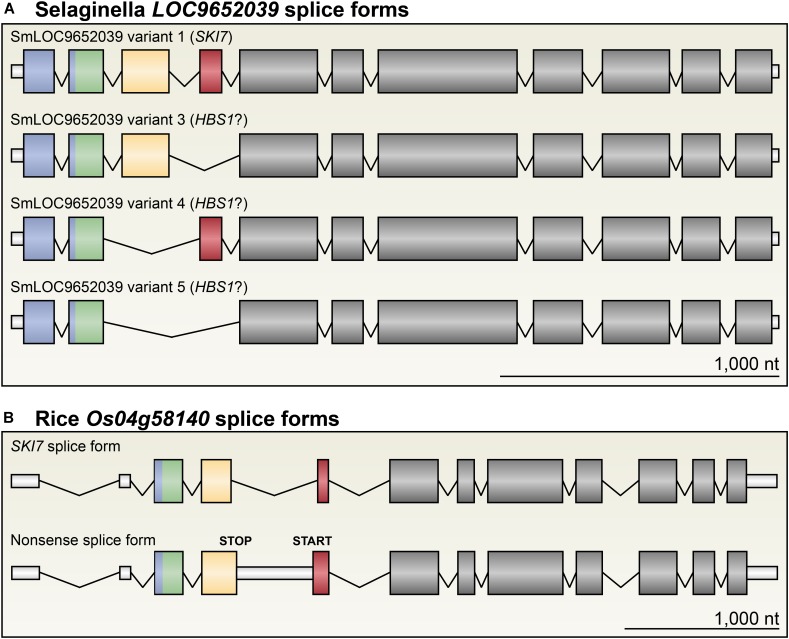
*SKI7* alternative splicing has distinct functional consequences in different plant linages. **(A)** *LOC9652039* in *S. moellendorffi* is alternatively spliced to either encode a complete SKI7 protein (variant 1), a putative HBS1 protein lacking the Patch 4-like motif (variant 3), the SKI7-like motif (variant 4), or both (variant 5). **(B)** *Os04g58140* is the only SKI7-encoding locus in rice. *Os04g58140* is alternatively spliced to retain intron 4, which includes several premature stop codons. Thus, this alternative splice form (bottom) does not encode a functional HBS1 or SKI7 protein, and is likely subject to NMD. As a result, unlike most other plant lineages examined, no single gene in rice can putatively encode both *HBS1* and *SKI7*.

In the genomes of *S. fallax*, *P. patens*, *A. trichopoda*, *M. truncatula*, and *T. cacao*, there are single-copy *HBS1/SKI7* orthologs that are each very similar to *At5g10630* (**Figures 2C**, **4A**, **5A**). Like in Arabidopsis, alternative splicing of transcripts from these loci can include or exclude a cassette exon that encodes the SKI7-like region (**Figures 2C**, **4A**). We therefore tentatively propose that this is the ancestral form of *HBS1/SKI7* in land plants, although as more deeply annotated transcriptomes of early-diverging land plant species become available, this proposal may require revision. *S. moellendorffi* encodes two *HBS1/SKI7* orthologs that generate several transcript permutations by alternative splicing (**Figure 6A**). Again, like Arabidopsis, alternative splicing of transcripts from these loci can include or exclude the SKI7-like motif. *LOC9640201* can also be alternatively spliced to remove both the SKI7-like and the Patch 4-like motifs. *LOC9652039* is even more complex: it can be alternatively spliced to remove only the SKI7-like motif (variant 4), only the Patch 4-like motif (variant 3), or both the SKI7-like and Patch 4-like motifs (variant 5) (**Figure 6A**). All of these alternatively spliced variants of *S. moellendorffi HBS1/SKI7* genes are predicted to encode proteins that function as HBS1, but not as SKI7.

In the *Solanaceae*, an ancestral gene duplication resulted in two distinct sets of orthologs (**Figure 4B**). In *S. lycopersicum*, one gene, *Solyc03g119290*, is similar to the typical land plant *HBS1/SKI7* orthologs, and is alternatively spliced to include or exclude the SKI7-like region (**Figure 4B**, upper panel). A second gene, *Solyc12g036410*, encodes regions that are highly homologous to HBS1, including the negatively charged N-terminus and C-terminal GTPase (**Figure 4B**, lower panel). The Zn-finger domain has tandemly duplicated, and both Zn-finger domains are on a cassette exon that is alternatively spliced (**Figure 4B**, lower panel; **Figure 5A**). The highly conserved Patch 4-like motif is absent in this gene (**Figure 4B**, lower panel; **Figure 5A**). A similar situation is found in a distantly related species of *Solanaceae*, *N. benthamiana*, which has two homeologous copies of the *At5g10630-/Solyc03g119290*-like gene, and one ortholog of the *Solyc12g036410* gene that has a duplicated Zn-finger domain and has lost the sequences to encode the SKI7-like and Patch 4 motifs (**Figure 5A**). Thus, duplication of the *HBS1/SKI7* locus in an ancestor of *Solanaceae* led to evolution of two distinct genes: one can encode HBS1 or SKI7-like orthologs, while the other has subfunctionalized to encode only an HBS1-like protein, and not SKI7 (**Figure 4B**).

The *HBS1/SKI7* orthologs are more diverse in the grasses, which have evolved distinct, subfunctionalized *HBS1* and *SKI7* loci. The grasses are divided into two major lineages: the PACMAD clade, which includes *Z. mays* (corn) and *S. viridis* (millet), and the BOP clade, which includes *O. sativa* (rice). Rice has three *HBS1/SKI7* orthologs: *Os04g50870*, *Os04g58140*, and *Os01g02720* (**Figures 5A**, **6B** and **Supplementary Data Sheet 2**). *Os04g50870* and *Os01g02720* are nearly identical paralogs (they encode proteins with 97% amino acid identity) that lack the SKI7-like and Patch 4-like motifs (**Figure 5**, “*Oryza HBS1a*” and “*Oryza HBS1b*”; **Supplementary Data Sheet 2**). *Os04g50870* and *Os01g02720* therefore most likely encode functional HBS1 proteins, but not functional SKI7 proteins. *Os04g58140* has two splice forms (**Figure 6B**). *Os04g58140.1* encodes a protein that includes the SKI7-like and Patch 4-like motifs (**Figures 5A**, **6B**). *Os04g58140.2* retains an intron that includes a premature stop codon and a downstream alternative start codon (**Figure 6B** and **Supplementary Data Sheet 2**). If the alternative start codon is selected (either by skipping the upstream open reading frame (uORF) or by reinitiating translation after the uORF), the Os04g58140.2 protein contains only the Patch 4-like motif and the HBS1-like translational GTPase. The *Os04g58140.2* transcript may also be subject to NMD, if the first start codon is selected and translation does not reinitiate at the alternative start codon. In either case, retention of the poison intron yields an unproductive transcript of *SKI7*.

In the PACMAD clade of grasses, *S. viridis* has two *HBS1/SKI7* orthologs, which have apparently subfunctionalized: *Sevir3g016200* encodes a complete SKI7-like protein, with the SKI7-like and Patch 4-like motifs, and *Sevir9g199100* encodes a protein with only the negatively charged N-terminus, the Zn-finger domain, a poorly-aligned/non-consensus Patch 4-like motif, and the C-terminal HBS1-like GTPase (**Figures 4C**, **5A** and **Supplementary Data Sheet 2**). RNA-Seq analysis suggests that neither of these transcripts is alternatively spliced, although there are relatively limited data for *S. viridis* compared to well-established model systems, like tomato and Arabidopsis. *Z. mays* has orthologs of both *S. viridis* genes (*Zm00001d026213* is orthologous to *Sevir9g199100*, and *Zm00001d001827* is orthologous to *Sevir3g016200*, **Figure 4C**), but its *SKI7-*like gene makes many distinct transcripts. Alternative 5′ and 3′ splice sites near the beginning of the coding sequence select between two different start codons, but only alter the N-terminus by eight amino acids. More importantly, some of these transcripts (such as *Zm00001d001827.2*) encode an entire SKI7-like protein, while others (such as *Zm00001d001827.9*) skip an exon that encodes the patch 4 motif (**Figure 4C**, lower panel), similar to *S. moellendorffi LOC9652039* variant 3 (**Figure 6A**).

### Defining Conserved SKI7 Protein Features by Homology

To assemble all reliable SKI7-like amino acid sequences, we queried the NCBI RefSeq_protein database^1^ for proteins with SKI7-like sequences in land plants and metazoans (**Supplementary Data Sheets 2**, **3**, respectively). Using this approach, we found a number of previously unidentified SKI7-like proteins in divergent invertebrate lineages, including orthologs in cnidarians, echinoderms, cephalochordates, mollusks, brachiopods, and priapulids (**Supplementary Data Sheet 3**). These findings suggest that SKI7-like orthologs are probably ubiquitous in eukaryotes, although deeper sequencing of transcriptomes from diverse phylogenetic clades will be needed to fully support this hypothesis, as well as to determine when the metazoan SKI7-like proteins lost the C-terminal HBS1-like GTPase and how the *HBS1/SKI7* gene/transcript structures evolved in the metazoans (**Figure 2A**).

**FIGURE 7:**
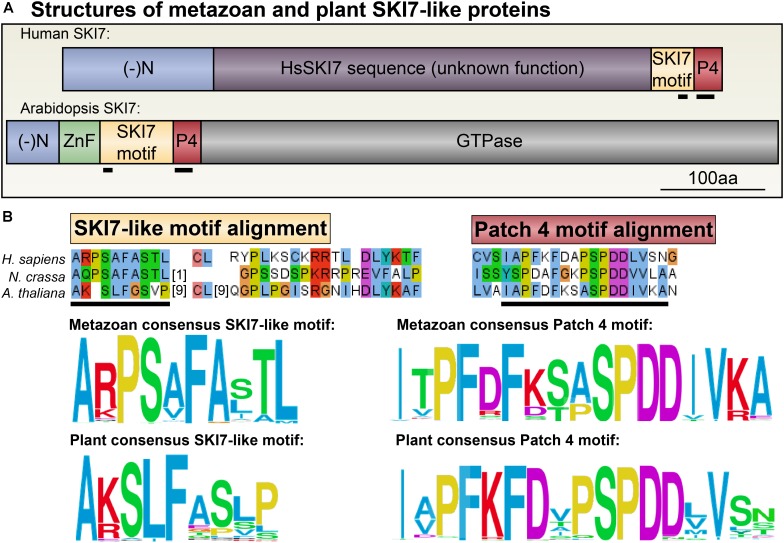
Consensus amino acid sequences that define eukaryotic SKI7. **(A)** Human and Arabidopsis SKI7 proteins do not globally align. Human SKI7 includes a long insert of unknown function (pink) between the negatively charged N-terminus (blue) and the SKI7- and Patch 4-like motifs (red). Arabidopsis SKI7 does not include this insert, but does include a C-terminal trGTPase domain of unclear function for SKI7, but which is required for HBS1 decoding activity. **(B)** Alignments of NCBI RefSeq SKI7 proteins from metazoans (**Supplementary Data Sheet 2**) and plants (**Supplementary Data Sheet 1**) were analyzed to determine consensus sequences for the SKI7-like motifs and the Patch 4-like motifs in these two major eukaryotic lineages. The sequences are largely similar, although the metazoan SKI7-like motif includes a widely conserved proline insertion (at position 3 in the consensus sequence). Alignments of the human, *Neurospora crassa* (fungus), and Arabidopsis SKI7-like motif and Patch 4-like motifs, as well as surrounding residues, are shown above the consensus sequences as examples. Amino acids are colored following Clustal standards, as above (**Figure 5A**).

We aligned SKI7-like sequences from metazoans and land plants to identify any conserved regions that could illuminate how sequences specific to SKI7, but not HBS1, determine its distinct functions (**Figure 7B** and **Supplementary Data Sheets 2**, **3**). There are two clearly conserved motifs across all SKI7 orthologs, which were previously named RxxxFxxxL and PFDFxxxSPDDIVKxNQ, based on the human SKI7-like protein sequence, and which we have named the SKI7-like motif and the Patch 4-like motif, respectively (**Figure 7**). Homology modeling of HsSKI7 onto the well-studied structure of the Rrp6/Rrp43 interaction revealed that the SKI7-like motif likely docks HsSKI7 with Rrp43, a core RNA exosome subunit (Kalisiak et al., 2017). The critical yeast SKI7-like motif residues, RxxxFxxxL, are not conserved across all eukaryotes, however; the consensus sequence at this site in metazoans is A+PShFAahL (where + is a positively charged residue, h is a hydrophobic residue, and a is an aliphatic residue; **Figure 7B**). In plants, this consensus sequence is slightly different: A+SLFaaa[P/L] (where + is a positively charged residue, a is an aliphatic residue, and [P/L] is usually P or L; **Figure 7B**). The Patch 4-like motif is fairly similar across all metazoans and plants. In metazoans, we found that the consensus Patch 4-like sequence is IaPF[D/R]F[K/D][S/T]aSPDDIV+A (**Figure 7B**). In plants, the consensus Patch 4-like sequence is IaPFKFDaPSPDDhVxx (**Figure 7B**). According to the resolved cryo-EM structure of yeast SKI7 in complex with the exosome (Kowalinski et al., 2016), the Patch 4-like motif mediates interactions with the Csl4 RNA exosome subunit. It should be noted that co-immunoprecipitation experiments in humans suggest that the Patch 4-like motif is neither necessary nor sufficient to recruit HsSKI7 to the RNA exosome, but the SKI7-like motif (A+PShFAahL) and neighboring residues were necessary and sufficient to recruit HsSKI7 or GFP to the RNA exosome (Kalisiak et al., 2017). Nonetheless, loss of the Patch 4-like motif did apparently weaken the interaction between SKI7 and the RNA exosome.

**FIGURE 8:**
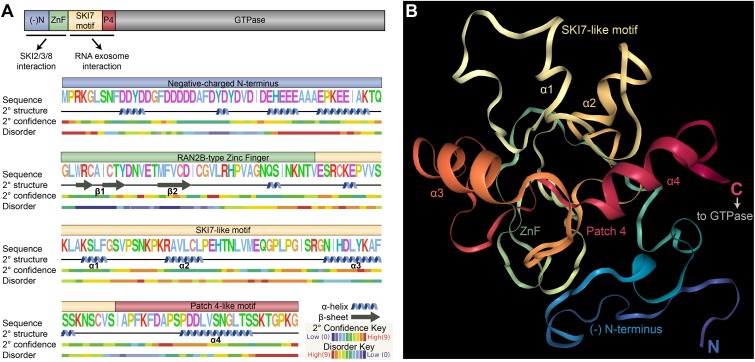
Predicted structure of the Arabidopsis SKI7 N-terminus. **(A)** Phyre2 prediction of the secondary structure and disorder of the N-terminal 183 amino acids of Arabidopsis SKI7. The negatively charged N-terminus (blue, (-)N) and RAN2B-type Zinc Finger (green, ZnF) are predicted to interact with the SKI2/SKI3/SKI8 cytosolic exosome adaptor complex, and the SKI7-like motif (yellow) and Patch 4-like motif (red, P4) are predicted to interact with the surface of the RNA exosome catalytic core. The negatively charged N-terminus includes moderate-confidence α-helices that may promote interaction with SKI2/SKI3/SKI8. The ZnF domain includes two β-sheets (β1 and β2) and an α-helix that coordinate with the Zn ion. The SKI7-like and Patch 4-like motif include four α-helices (α1 through α4), which is structurally comparable to the yeast SKI7 protein. **(B)** Phyre2 prediction of the structure of the N-terminal 183 amino acids of Arabidopsis SKI7, based on homology modeling against several resolved protein structures (as described in the methods). Residues are colored by position, from blue (N-terminus) to red (C-terminus), closely matching the colors used in panel a and other figures. The defined regions of the protein are labeled, including α1, α2, α3, and α4, which are predicted to mediate interactions with the RNA exosome core. The C-terminus of this structure is predicted to be highly disordered, forming a flexible linker to the C-terminal trGTPase of Arabidopsis SKI7.

We used homology modeling to predict the structure of the N-terminus of the Arabidopsis SKI7 protein (excluding the C-terminal trGTPase, **Figure 8**). The negatively charged N-terminus is predicted to form several α helices, which may mediate interactions with the SKI2/SKI3/SKI8 adaptor complex, and the ZnF is predicted to form two β-sheet-like structures (β1 and β2, **Figure 5A**) followed by an α-helix, as expected for a ZnF domain. The exons encoding the SKI7-like motif and Patch 4-like motif fold into four α-helices (α1 through α4, **Figure 5A**), very comparable to the resolved yeast SKI7 structure (Kowalinski et al., 2016). α1 overlaps with the deeply conserved SKI7-like motif (in Arabidopsis, AKSLFGSVP, **Figure 8A**). α3 is highly similar between humans and Arabidopsis (**Figure 8A**), including residues DLYKAF (Arabidopsis) or DLYKTF (human), and this α-helix has been labeled in the predicted Arabidopsis SKI7 structure (**Figure 8B**). The Patch 4-like motif is predicted to be highly structured, and forms α4. Immediately after α4, the last amino acids of this N-terminal region of Arabidopsis SKI7 are highly disordered (this pattern continues into the N-terminal residues of the trGTPase region of the protein), allowing the trGTPase to adopt a flexible position relative to the highly structured N-terminus that interacts with the RNA exosome (**Figure 8B**).

## Discussion

**FIGURE 9:**
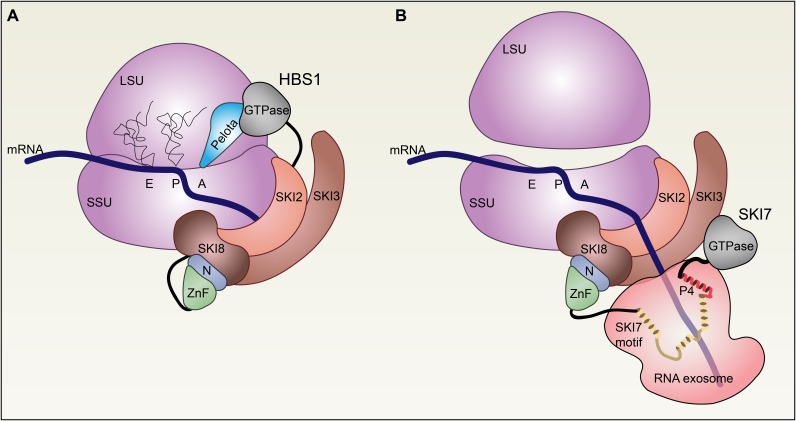
Model of the roles of HBS1 and SKI7 in mRNA surveillance and degradation. **(A)** HBS1 associates with PELOTA (light blue) to recognize stalled ribosomes (purple). The SKI2/SKI3/SKI8 complex (browns) interacts directly with the small subunit (SSU) of the 80S ribosome, perhaps facilitated by the HBS1 N-terminal domains (green and blue), which are known to interact with SKI3 and SKI8. Structural models suggest that PELOTA displaces mRNA from the aminoacyl A site, allowing the mRNA to interact with the SKI2 RNA helicase. **(B)** PELOTA and HBS1 promote 80S ribosome dissociation from the transcript and splitting into the 60S (large subunit, LSU) and 40S (SSU) subunits. SKI7 recruits the RNA exosome 3′→5′ exoribonuclease complex (light red) to SKI2/SKI3/SKI8 via interactions between the SKI7 N-terminus and SKI3/SKI8. α helices in the SKI7-like motif (yellow) and Patch 4 (P4)-like motif (red) form an interaction surface with the RNA exosome complex.

Here, we have shown that *HBS1/SKI7* is a well-conserved locus in eukaryotes that encodes two proteins with distinct molecular functions. In plants and fungi, HBS1 and SKI7 are nearly identical proteins, with an N-terminus that interacts with the cytosolic RNA exosome SKI2/SKI3/SKI8 complex and a C-terminal translational GTPase. The SKI7 isoform differs from HBS1 by as few as ∼25 amino acids that we propose promote its interaction with the RNA exosome instead of with the ribosome decoding factor, PELOTA (**Figure 9**).

Although we have shown that many plant genomes encode both HBS1 and SKI7 from a single locus by alternative splicing of an exon encoding the SKI7-like motif, other lineages have evolved distinct *HBS1/SKI7* gene structures. In the early-diverging tracheophyte *S. moellendorffi*, *HBS1/SKI7* can be alternatively spliced to exclude or include exons encoding either the SKI7-like motif or the Patch 4-like motif, or to exclude or include both of these exons. In the *Solanaceae*, a second *HBS1* locus has lost the SKI7-like and Patch 4-like motifs, and thus encodes only an HBS1-like protein. In rice, HBS1 and SKI7 are each encoded by their own locus, reminiscent of the subfunctionalization of *Hbs1* and *Ski7* loci in *S. cerevisiae*. Panicoid grasses (maize and millet), like rice, have two distinct *HBS1* and *SKI7* loci: one encodes only HBS1, and the second can encode SKI7. The panicoid grass *SKI7* locus is alternatively spliced to include or exclude the Patch 4-like motif, however, which may impact its function. The Patch 4-like motif is universally conserved in all known SKI7 orthologs, but is not conserved in HBS1 orthologs that have lost other SKI7-like features (e.g., the HBS1-specific loci in *Solanaceae*, rice, and yeast), suggesting that the Patch 4-like motif is critical for SKI7’s functions. In humans, however, Patch 4-like is neither necessary nor sufficient to recruit SKI7 to the RNA exosome (Kalisiak et al., 2017). Thus, while it seems most likely that the panicoid grass SKI7-like protein loses functionality when the exon encoding the Patch 4-like motif is excluded by alternative splicing, this will need to be determined experimentally.

Consideration of the evolutionary history of *HBS1/SKI7* loci in eukaryotes reveals an important distinction: subfunctionalized *HBS1* paralogs that cannot encode SKI7 have evolved repeatedly, and are often no longer regulated by alternative splicing, but in almost all eukaryotes (except for *S. cerevisiae* and a handful of other fungi), *SKI7* orthologs are alternatively spliced (Marshall et al., 2013; Lambert et al., 2015). Moreover, alternative splicing of *SKI7* consistently has strong effects, either yielding an unproductive splice form that is likely degraded by NMD, a splice form that encodes a loss-of-function protein, or a transcript that instead encodes HBS1. This consistent regulation of SKI7 levels by alternative splicing suggests that the activity of the cytosolic RNA exosome is tightly coordinated with nuclear RNA processing, especially conditions that shift alternative splicing dynamics, such as oxidative stress (Staiger and Brown, 2013; Berner et al., 2017). Further characterization of the developmental or physiological conditions that influence alternative splicing of *SKI7*, as well as assays to determine whether SKI7 protein levels are limiting factors in cytosolic RNA exosome activity, will be needed to unravel how this mechanism influences cytosolic RNA exosome activity. Recently, the Pelota/HBS1 decoding factors and the cytosolic RNA exosome have been implicated in promoting NMD (Arribere and Fire, 2018), and NMD is known to regulate expression of the splicing and translation machinery (Lareau and Brenner, 2015). Our finding that alternative splicing of *SKI7* potentially regulates SKI7 levels to limit assembly of the SKI2/SKI3/SKI8-RNA exosome complex (by either excluding an alternative exon of *SKI7* to encode HBS1, or by generating a nonsense *SKI7* transcript) invites speculation that SKI7 regulates its own splicing and transcript stability via its role in NMD and NMD-mediated regulation of splicing machinery gene expression.

PELOTA and the cytoplasmic RNA exosome are emerging as crucial components of plant immune systems, although their necessary interacting partners, HBS1 and SKI7, have not been comprehensively defined in plants until now. Loss of PELOTA, the decoding factor that recruits HBS1 to stalled ribosomes, confers resistance to TYCLV infection in tomato; with our identification of tomato *HBS1* and *HBS1/SKI7* genes, it is now possible to test whether loss of HBS1, SKI7, and/or SKI2/SKI3/SKI8 also confer resistance to TYCLV, and how this RNA degradation machinery interacts with other viruses. In rice, a recessive *pelota* mutant triggers a salicylic acid-associated autoimmune response, including spontaneous lesions and dwarfism, through unclear mechanisms (Ding et al., 2018; Qin et al., 2018; Zhang et al., 2018). The rice *pelota* defects could be related to hyperaccumulation of aberrant RNA species, similar to the tricohepatoenteric autoimmune syndrome in *skiv2l* human cells (Eckard et al., 2014), or due to specific dysregulation of transcripts in rice that regulate immunity. For instance, the expression and activity of the disease resistance Toll- and Interleukin-like Receptor (TIR) family of Nucleotide-binding, Leucine-rich repeat Receptors (TIR-NLRs or TLRs) is regulated by NMD in some instances; loss of PELOTA, which contributes to NMD, may therefore deregulate *TLR* expression, triggering autoimmune defects (Dinesh-Kumar and Baker, 2000; Riehs-Kearnan et al., 2012; Gloggnitzer et al., 2014). Whether *pelota* mutants can trigger autoimmune defects in other plant species remains to be determined. More broadly, NMD is proposed as a general antiviral mechanism, and so HBS1 and SKI7 may contribute to broad-spectrum antiviral defense via their roles in NMD (Balistreri et al., 2014; Garcia et al., 2014; Rigby and Rehwinkel, 2015; Hamid and Makeyev, 2016).

## Conclusion

We have identified the plant orthologs of HBS1 and SKI7, key regulators of RNA metabolism in eukaryotic cells. As a component of the cytosolic RNA exosome, SKI7 not only participates in co-translational RNA surveillance, but is also presumably required to clear 5′ fragments of mRNAs cleaved by RISCs. RNA exosomal degradation of these 5′ fragments prevents copying of host transcripts by RDR6, which can otherwise trigger post-transcriptional silencing of endogenous genes. In diverse eukaryotic lineages, SKI7 levels are controlled by alternative splicing of transcripts; alternative splice forms can either encode the functionally distinct HBS1 protein, or can be unproductive, either by removing critical residues for SKI7 function, or by introducing premature stop codons that likely subject the splice form to NMD. Co-translational RNA decay mechanisms, including HBS1/SKI7-dependent RNA degradation, are becoming more prominent to investigations of eukaryotic immune systems and defenses against viral infection. Our discovery of the alternative splicing of *HBS1/SKI7* expression across anciently diverging eukaryotic lineages, including plants and invertebrate clades, implies that co-translational RNA decay mechanisms are under complex regulation to coordinate host gene expression with environmental cues, stress responses, and antiviral defense.

## Materials and Methods

### Plant Materials

The Landsberg *erecta* (L*er*) ecotype of Arabidopsis was grown under 16 h light (100 μmol photons m^-2^ s^-1^ of photosynthetically active radiation)/8 h dark cycles. Shoots were harvested 4 weeks after germination.

### RT-PCR and TOPO Cloning

RNA was isolated from L*er* Arabidopsis plants with the Spectrum Plant Total RNA (Sigma-Aldrich) kit with on-column DNase I digestion (New England Biolabs). cDNA was synthesized from RNA using oligo (dT)18 primers and SuperScript III reverse transcriptase (Fisher Scientific). Splice forms were amplified with Phusion DNA polymerase (New England Biolabs), adding a CACC 5′ overhang to facilitate pENTR/D-TOPO cloning. RT-PCR amplified DNA was gel purified in a 1% agarose gel and extracted using a gel extraction kit (Bioneer). Purified DNA was used for TOPO reactions with pENTR/D-TOPO (Thermo Fisher), transformed into XL1-Blue *E. coli* chemically competent cells, and screened for resistance to kanamycin on LB agar. Plasmid was purified from positive colonies using a miniprep kit (Bioneer) and sequenced using Sanger technology with the M13F primer.

Oligonucleotides used for cloning were: JB1058: 5′-CACC ATG CCT CGT AAA GGA TTA TCC AAT TTC G-3′, JB1061: 5′-CACC ACA GTT GAG AGCAG ATG CAA AGA AC-3′, and JB1063: 5′-GCC TTT TGG ACC AGT TTT TGAGG ATG-3′. JB1058 + JB1063 surround the alternative exon, and amplified three majors products: the short splice form, the long splice form, and a small amount of the longer, minor splice form. JB1061 is specific to the 5′ end of the alternative exon, and in combination with JB1063, amplified both the long splice form and the longer, minor splice form.

### Computational Analysis

Decoding trGTPases were identified using human protein sequences as queries for a BLASTp search against the *Arabidopsis thaliana* refseq protein database. Putative trGTPases were filtered to include only cytosol-localized proteins, based on proteomic data and consensus predictions curated by the Subcellular Localization Database for Arabidopsis Proteins 3^2^.

SKI7 orthologs in the NCBI protein refseq databases were identified using a tBLASTx search with the Arabidopsis SKI7-like and patch 4-like motifs as a query for land plants, and the human SKI7-like and patch 4-like motifs as a query for metazoans. BLASTp results were then filtered to remove identical protein sequences. Protein sequences were aligned using MAFFT via JalView. Uncommon insertions were trimmed from the final alignments for clarity. Transcript structures were obtained for the select plant species described in the text from relevant databases (TAIR10, from arabidopsis.org and araport.org; MaizeGDB.org; Phytozome.jgi.doe.gov; SolGenomics.net; and CosMoss.org), and then confirmed with RNA-Seq evidence from the same databases (or by direct cloning of alternative splice forms, as described above). Consensus sequence logos were generated with WebLogo^3^.

The N-terminus of SKI7 (through the patch 4-like motif) structure was modeled by Phyre2^4^, which used structures of YY1-associated factor 2 (PDB 2D9G), HBV-associated factor (PDB 2CRC), Rubredoxin B (PDB 2KN9), NEMO CoZi (PDB 4OWF), and TAB3-NZF (PDB 3A9K). The resulting model was visualized by NGL^5^.

## Author Contributions

JB designed the project, conducted the experiments, and drafted the manuscript. BB contributed to the experimental design and manuscript.

## Conflict of Interest Statement

The authors declare that the research was conducted in the absence of any commercial or financial relationships that could be construed as a potential conflict of interest.

## Footnotes

1 https://www.ncbi.nlm.nih.gov/refseq/

2 suba3.plantenergy.uwa.edu.au

3 http://WebLogo.berkeley.edu

4 http://www.sbg.bio.ic.ac.uk/∼phyre2

5 http://proteinformatics.charite.de/ngl-tools/ngl/html/ngl.html
